# RETRACTION: Effects of Fast‐Track Recovery Programme on the Surgical Site Wound Infection in Patients Undergoing Biliary Stones Surgery: A Meta‐Analysis

**DOI:** 10.1111/iwj.70287

**Published:** 2025-03-03

## Abstract

**RETRACTION:** ZhouA. , LvX. , WangL. , WangH. , and LuM. , “Effects of Fast‐Track Recovery Programme on the Surgical Site Wound Infection in Patients Undergoing Biliary Stones Surgery: A Meta‐Analysis,” International Wound Journal 21, no. 1 (2024): e14546, 10.1111/iwj.14546.38272815 PMC10794056

The above article, published online on 17 January 2024, in Wiley Online Library (http://onlinelibrary.wiley.com/), has been retracted by agreement between the journal Editor in Chief, Professor Keith Harding; and John Wiley & Sons Ltd. Following an investigation by the publisher, all parties have concluded that this article was accepted solely on the basis of a compromised peer review process. The editors have therefore decided to retract the article. The authors did not respond to our notice regarding the retraction.

**RETRACTION:** A. Zhou , X. Lv , L. Wang , H. Wang , and M. Lu , “Effects of Fast‐Track Recovery Programme on the Surgical Site Wound Infection in Patients Undergoing Biliary Stones Surgery: A Meta‐Analysis,” International Wound Journal 21, no. 1 (2024): e14546, 10.1111/iwj.14546.38272815 PMC10794056

The above article, published online on 17 January 2024, in Wiley Online Library (http://onlinelibrary.wiley.com/), has been retracted by agreement between the journal Editor in Chief, Professor Keith Harding; and John Wiley & Sons Ltd. Following an investigation by the publisher, all parties have concluded that this article was accepted solely on the basis of a compromised peer review process. The editors have therefore decided to retract the article. The authors did not respond to our notice regarding the retraction.

